# Supplementary material for: Disruption of fos causes craniofacial anomalies in developing zebrafish
Source: Front Cell Dev Biol. 2023 Aug 16;11:1141893. doi: 10.3389/fcell.2023.1141893 (PMC10469461; doi:10.3389/fcell.2023.1141893)
Supplement: Supplementary file 1 [file Presentation1.pdf]

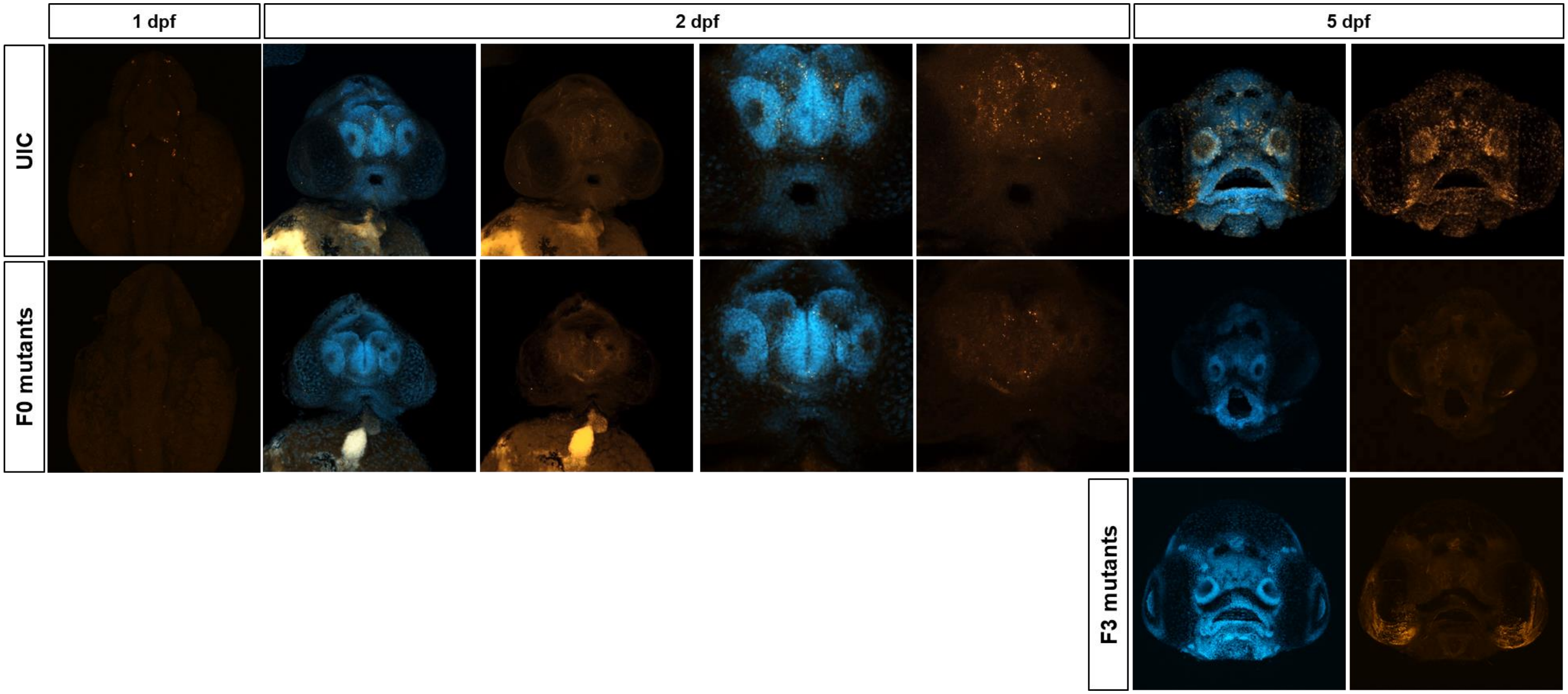

Supplemental Figure 1. ***Fos* expression in controls and *fos* F0 mutants (crispants).** Hybridization chain reaction (HCR) in situ with zebrafish *fos* mRNA probe set showed that *fos* is regionally expressed at 1 dpf (dorsal view shown). Expression was reduced in *fos* crispants at all developmental time points evaluated compared to controls. Stable F3 mutants showed absent *fos* mRNA expression at 5 dpf. UIC, uninjected control; F0, crispant; MO, morphant; MM, mismatch control morpholino

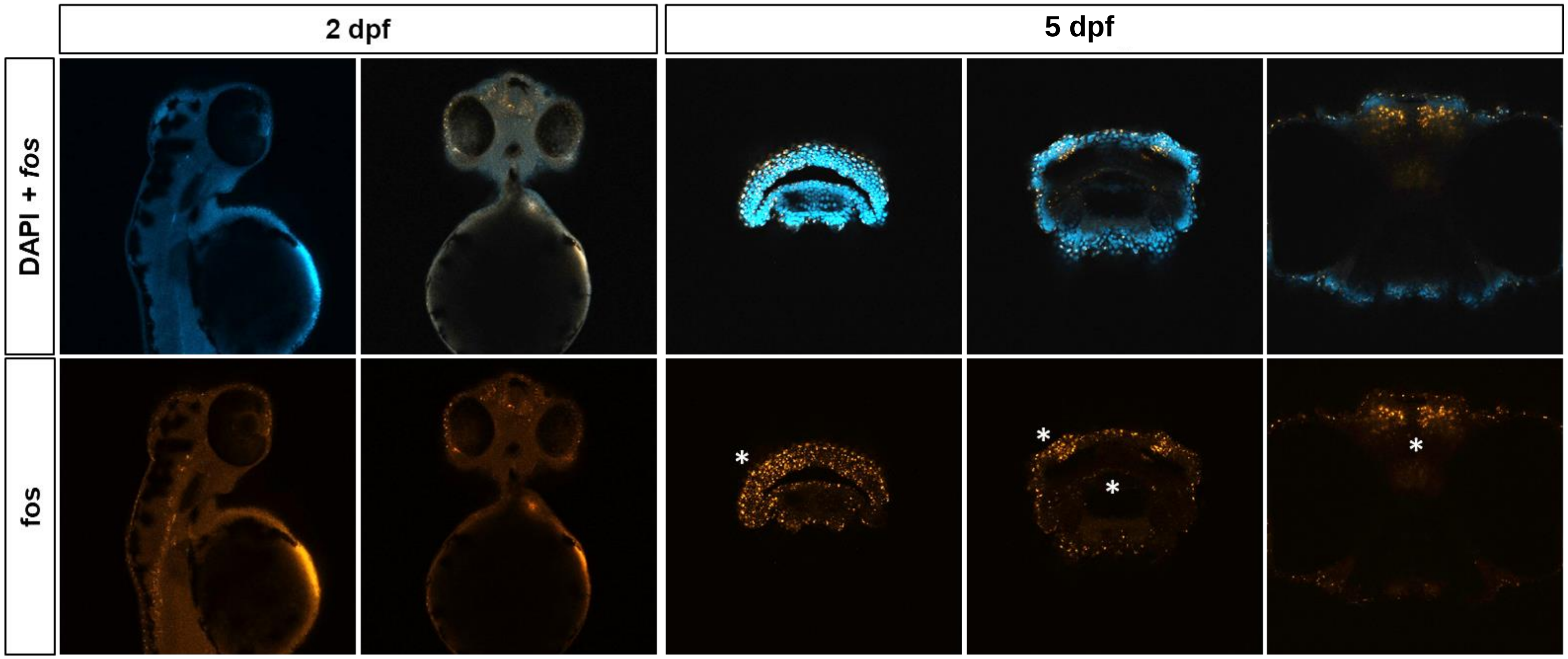

Supplemental Figure 2. Examination of individual confocal sections showed *fos* mRNA expression both in the inner as well as more superficial tissues at both 2 and 5 dpf. Interestingly, at 5dpf, strong expression was found in the brain (slice 45/60), palate and olfactory placodes (slice 17/60), and periderm (slice 4/60) (asterisks).

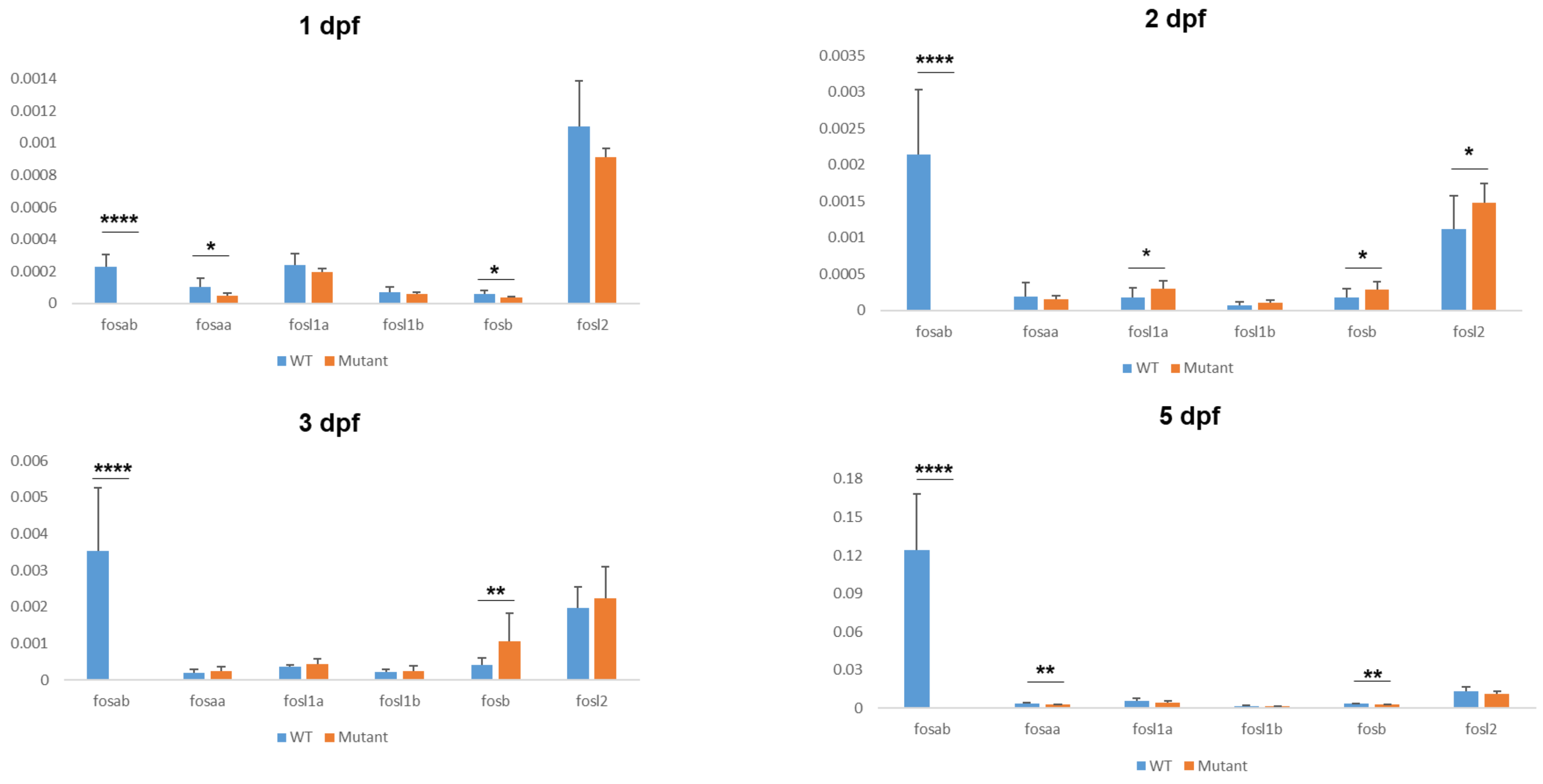

Supplemental Figure 3. RNA transcripts of *fos*-related genes in *fos* mutant embryos at 1-5 dpf. Student's t-test * $p < 0.05$, ** $p < 0.01$, *** $p < 0.001$, **** $p < 0.0001$. WT, wild type; mutant, *fos* stable mutant.

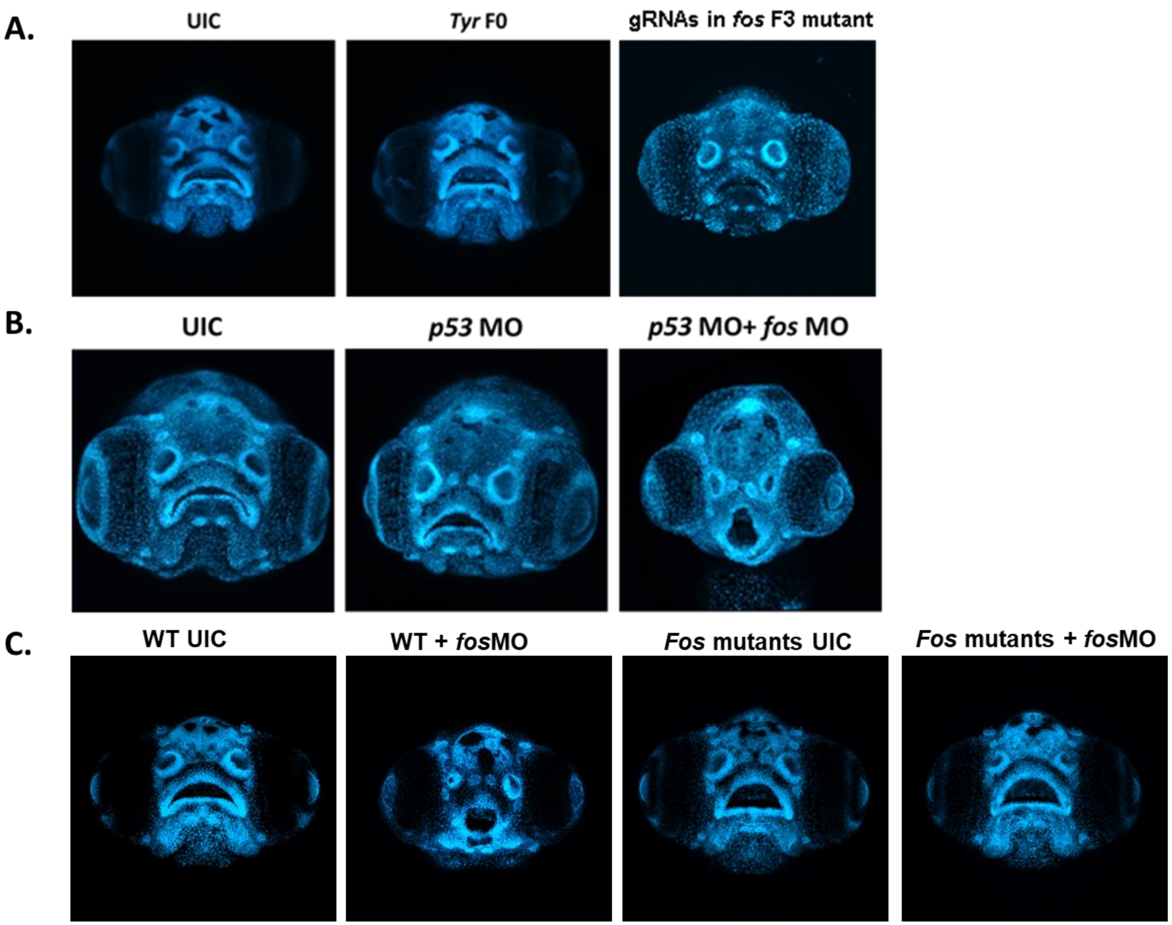

Supplemental Figure 4. Phenotypic effects are specific to *fos* perturbation. A) Tyrosinase control crispants (tyr F0) have a normal orofacial phenotype as do F3 mutants injected with the same crispr guides. **B**) *Fos* morphant phenotype is not rescued by p53 morpholino co-injection, indicating that p53-related neurotoxicity is not contributing to the observed craniofacial abnormalities in *fos* morphants. **C)** Injection of *fos* MO in stable fos F3 mutants did not lead to a craniofacial phenotype, further supporting *fos* MO specificity. Approximately 50 1-cell stage embryos were injected and a minimum of n = 10 were imaged for each condition. UIC, uninjected control, tyr, tyrosinase guide RNA (gRNA) injected; MO, morpholino;

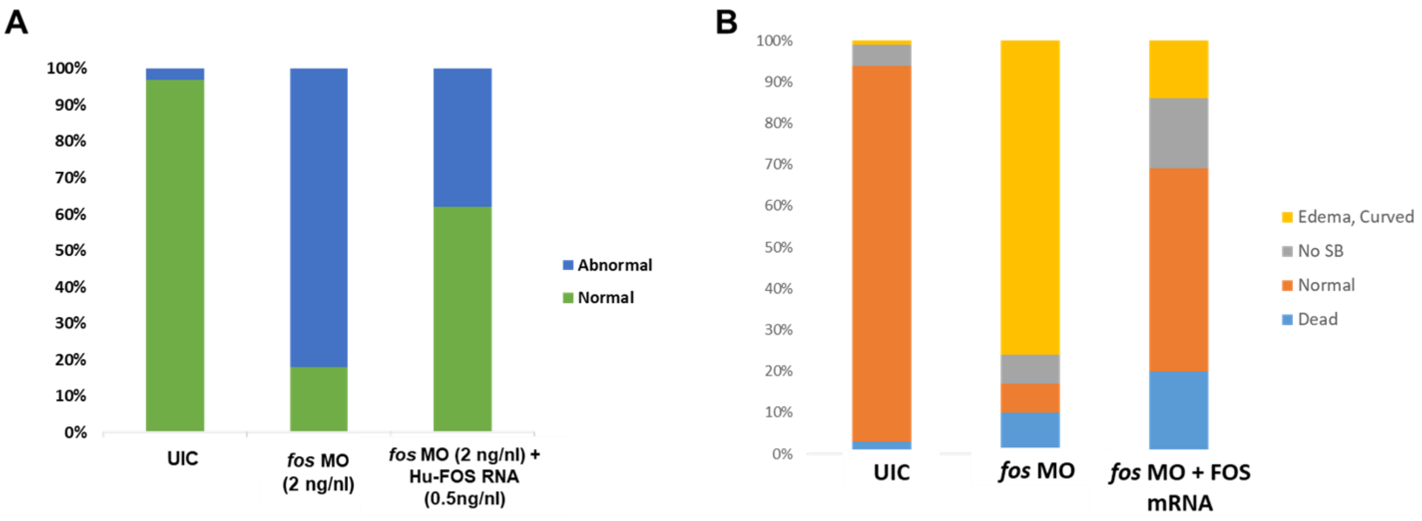

Supplemental Figure 5. ***Fos* morphant phenotype was rescued with human *FOS* mRNA injection. A)** Human *FOS* mRNA (Hu-FOS RNA) co-injection rescue decreased abnormal embryos by 45%. **B**) The edema and curved body phenotypes were also rescued by co-injection. Approximately 50 1-cell stage embryos were injected and a minimum of n = 20 were imaged and phenotypically examined for each condition. UIC, uninjected control; MO, morphant.

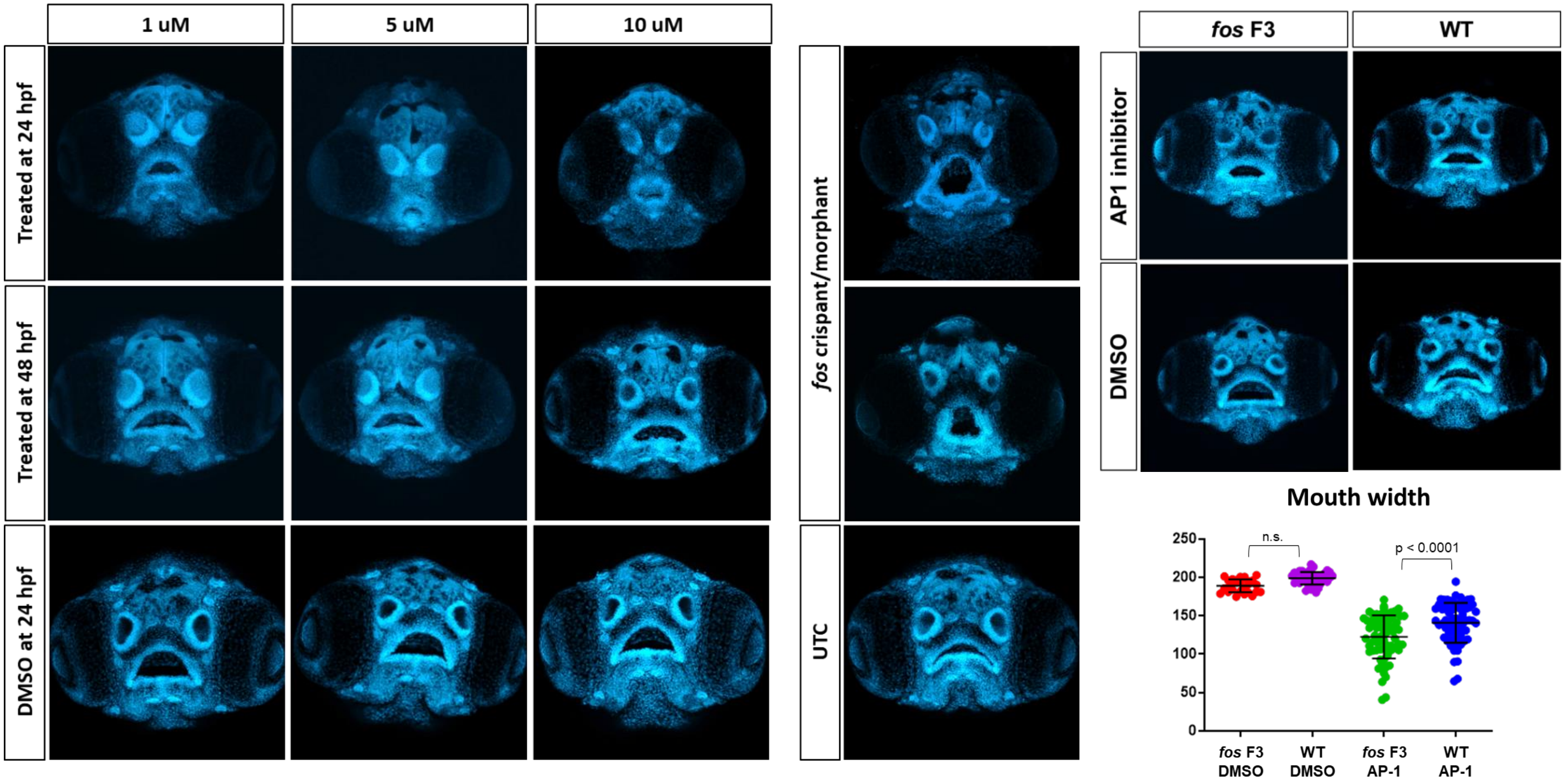

Supplemental Figure 6. **AP-1 inhibitor treatment alters craniofacial morphology.** Embryos were continuously treated with SR 11302 starting at 24 and 48 hpf and collected at 5 dpf. Dishes of n=50 larvae were treated for each condition and a minimum of n=15 for each condition were imaged. Earlier treatment at all doses led to more anomalies, including a horizontally constricted face, small mouth, abnormal nasal pits and merged neuromasts. When treatment was started at 48 hpf and a concentration of 10 uL, mouth shape was the most severely affected while at a concentration of at 1 uM the mouth was normal. Interestingly, when stable fos F3 mutants were treated with AP-1 inhibitor, they showed a more severe phenotype Test and p value (significantly decreased mouth width measurement is shown) suggesting they are more sensitive to AP-1 perturbation. The number of zebrafish analyzed was: drug treated fos F3 mutants= 70, drug treated wild type (WT) controls = 68, DMSO treated mutants = 29, DMSO treated controls = 40. UTC, untreated control; DMSO, vehicle control.

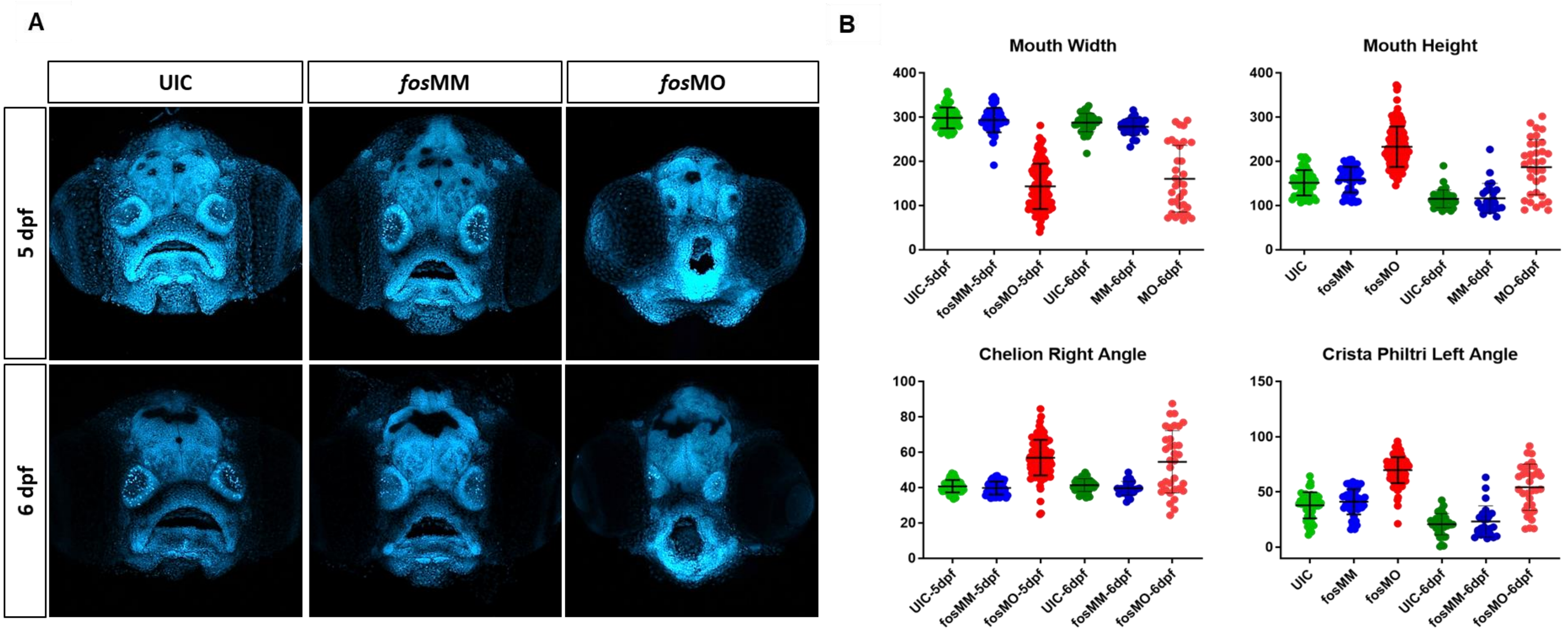

Supplemental Figure 7. ***Fos*** **morphants have altered mouth measurements at 6 dpf. A**) Representative DAPI images of 5 and 6 dpf embryos for each condition. **B**) *Fos* knockdown perturbs facial development with altered measurements for mouth width, mouth height, chelion right and crista philtra left angles, among others, at both 5 and 6 dpf, supporting that the facial phenotype is not due to developmental delay. ANOVA and p values The number of embryos analyzed were as follows: 5dpf- UIC = 98, MM = 113, MO = 138, 6dpf- UIC = 38, MM = 26, MO = 35. UIC, uninjected control; MO, *fos* morpholino, MM, mismatch control morpholino.

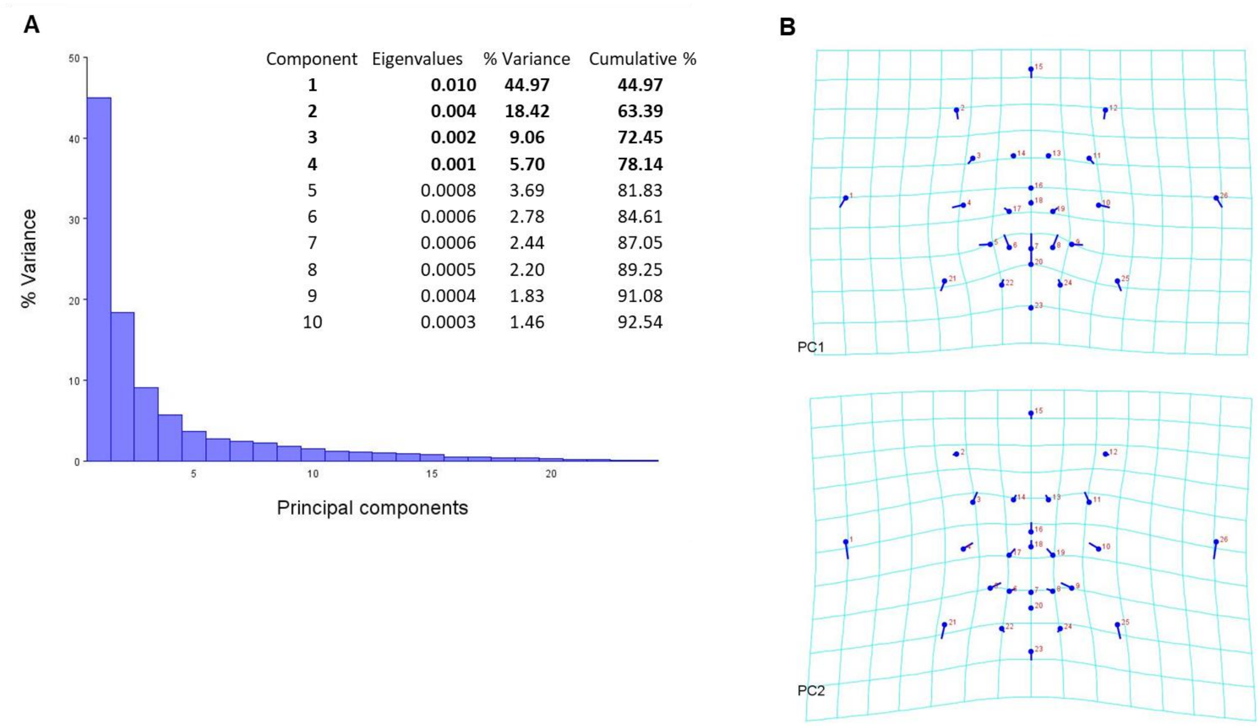

Component	Eigenvalues	% Variance	Cumulative %
**1**	**0.010**	**44.97**	**44.97**
**2**	**0.004**	**18.42**	**63.39**
**3**	**0.002**	**9.06**	**72.45**
**4**	**0.001**	**5.70**	**78.14**
5	0.0008	3.69	81.83
6	0.0006	2.78	84.61
7	0.0006	2.44	87.05
8	0.0005	2.20	89.25
9	0.0004	1.83	91.08
10	0.0003	1.46	92.54

Supplemental Figure 8. **Principal component analysis results after Procrustes transformation. A)** Scree plot depicting eigenvalues of all principal components (PCs), with the first 4 explaining 78% of the variance in the combined dataset. **B**) Transformation grids with lollipop graphs showing vectors of change for each landmark along the first two components, PC1 and PC2. The number of embryos analyzed in each group was untreated controls, n=93, *fos* F0 mutants n=103, *fos* morphants n=138, and mismatch morpholino controls, n = 47.

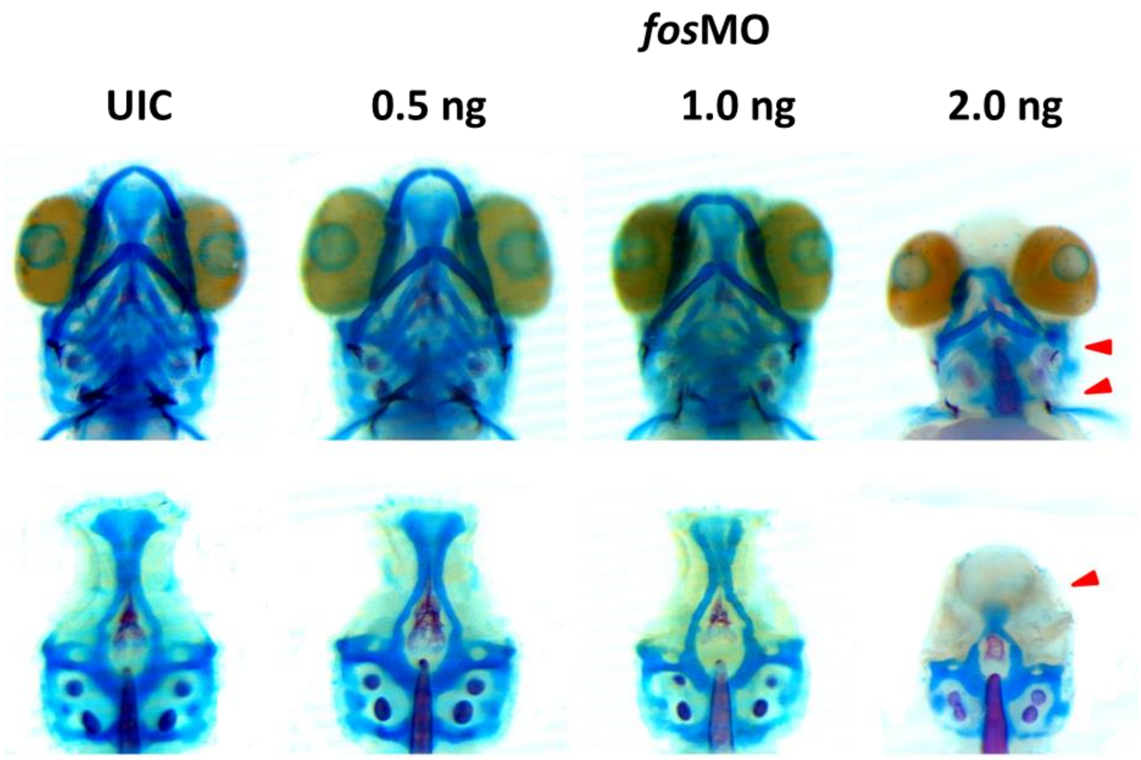

Supplemental Figure 9. Bone and cartilage phenotype severity increases with higher doses of *fos* morpholino. At the highest dose, embryos displayed a severely reduced primary palate, missing lower jaw cartilages and lack of pharyngeal teeth (red triangles). A minimum of n=50 1-cell stage embryos were injected for each dose and stained and n>=10 embryos were dissected and imaged for upper and lower jaw skeletal examination. UIC, uninjected control; MO, *fos* morpholino

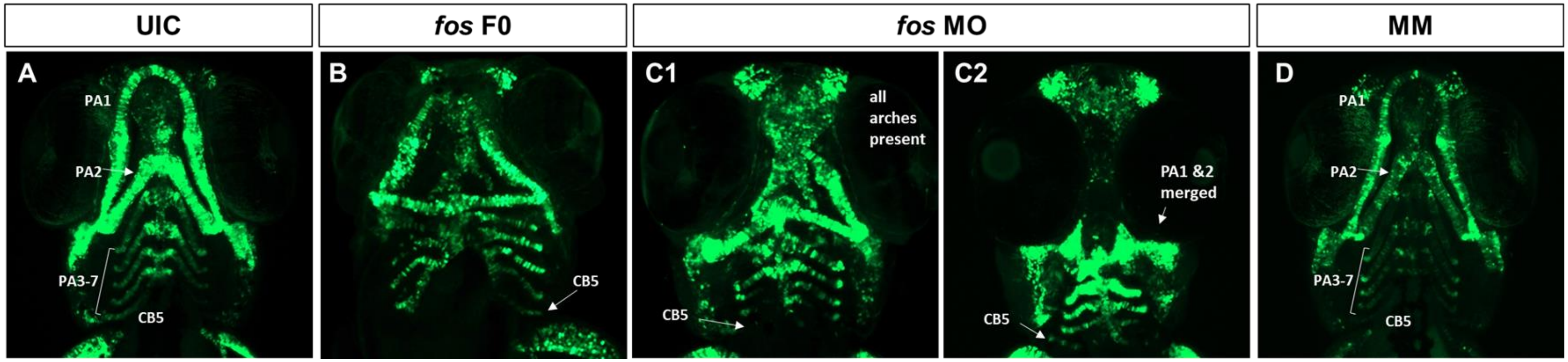

Supplemental Figure 10. **A**) Pharyngeal arches PA 1-7 at 5dpf in UIC. **B**) Abnormal arches in *fos* F0 mutants. **C**) The range of abnormalities observed in morphants compared to **D**) MM controls. UIC, uninjected control; F0, crispant; MO, morphant, MM, mismatch morpholino control. A minimum of n=28 zebrafish were imaged for this analysis.

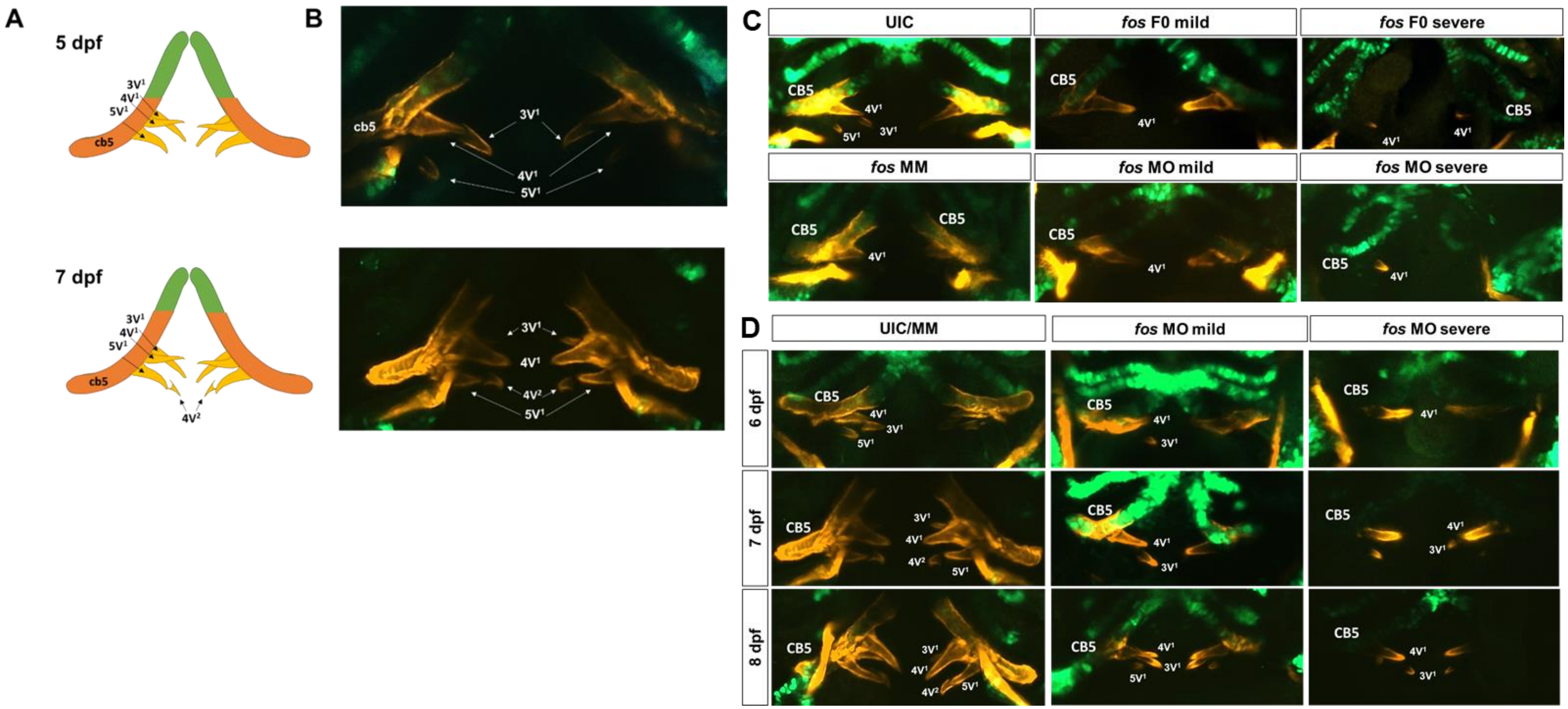

Supplemental Figure 11. Zebrafish pharyngeal tooth development is affected in *fos* morphants. **A**) Schematic representation of the 5th ceratobranchial arch (CB5) and pharyngeal teeth (3V1, 4V1, and 5V1) at 5 and 7dpf and **B**) confocal images of Alizarin red stained embryos at the same time points. **C**) *Fos* mutants and morphants were examined at 5 dpf and the range of tooth phenotypes is represented for each group. The number of zebrafish analyzed was UIC n=9, F0 n=10, MO n=42, MM n=15. **D**) Morphants were followed up to 8 dpf (5dpf UIC= 18, MO=84, MM =30; 6dpf UIC=8, MO= 60, MM =8; 7dpf UIC=22, MO=24, MM=16; 8dpf UIC=15, MO = 58, MM=10); defects in tooth attachment to CD5 and in tooth morphogenesis were observed throughout developmental stages. UIC, uninjected control; F0, crispant; MO, morphant, MM, mismatch morpholino control.

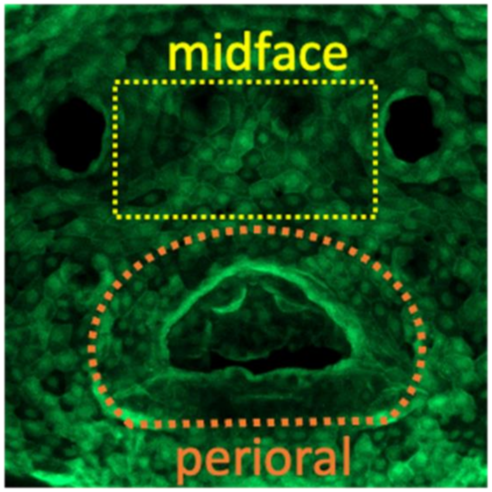

Supplemental Figure 12. Quantitation of periderm cell size. Schematic of regions where cells were measured. Cell size was compared for all 4 groups (UIC, crispant, morphant and MM control) at 3 and 5 dpf. Midface and perioral cells were analyzed separately due to existing differences in cell size.

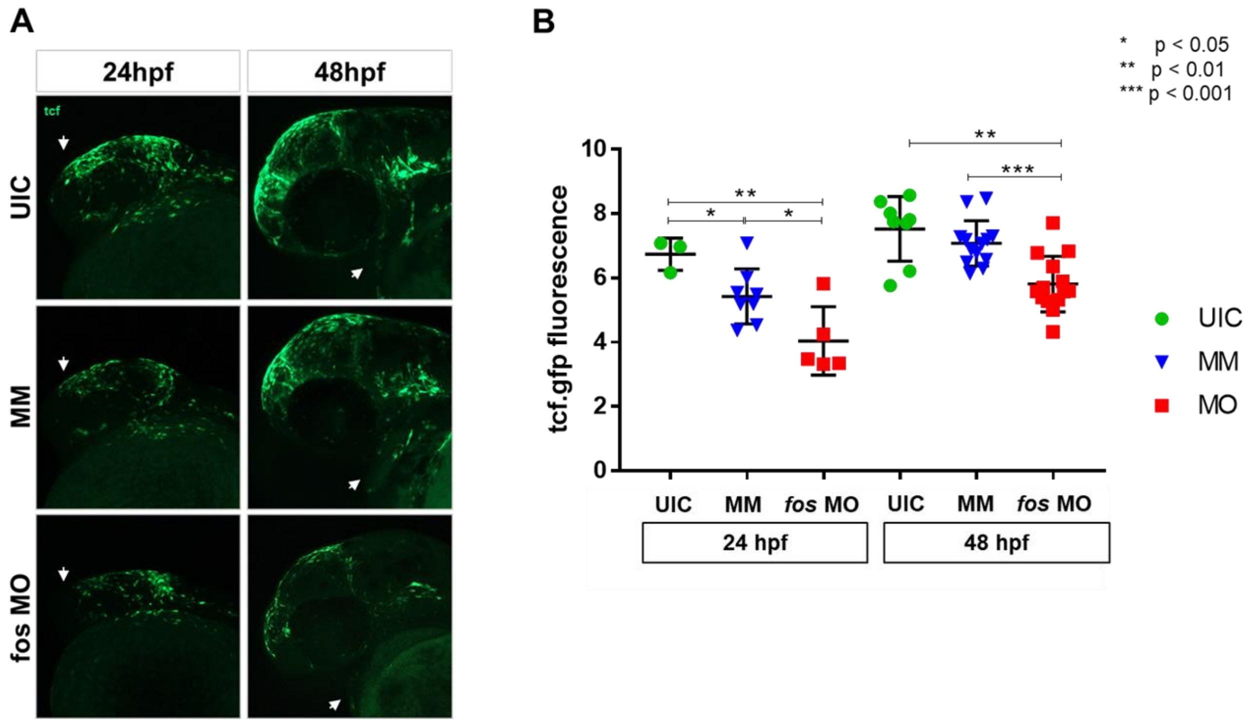

Supplemental Figure 13. Wnt/β-catenin pathway activation is significantly reduced in *fos* morphants. A) *Fos* knockdown in a Wnt biosensor *Tg(7xTCF-Xla.Siam:GFP)* line showed significant reduction of β-catenin responsive cells (arrows) at 24 and 48 hpf in *fo*s morphants as compared to MM and UIC. **B**) Fluorescence quantification using ImageJ showed significantly reduced gfp signal in *fos* morphants at both time points. Test and p-values The number of zebrafish analyzed was 24hpf UIC =3, MM=9, MO=5; 48hpf UIC=8, MM=14, MO=13. UIC, uninjected control; MO, morphant; MM, mismatch morpholino control.

Appendix: Landmarks and measurements used in morphometric analysis.

Feature Name	Landmarks used in measurement
Olfactory Distance	3 to 11
Upper Lip Width	16 to 18
Lower Lip Width	7 to 20
Mouth Width	5 to 9
Olfactory to Mouth	11 to 3 to 16
Olfactory to Mouth 2	3 to 11 to 16
Difference	
Olfactory to Mouth 3	3 to 16 to 11
Chin Width	21 to 25
Mouth to Chin	16 to 23
Alternate Height	Midpoint of 2 to 12 to point 23
Mouth Height	16 to 20
Neuromast Angle 1	2 to 4 to 16
Neuromast Angle 2	12 to 10 to 16
Neuromast Height	Midpoint of 2 to 12 to midpoint of 4 to 10
Neuromast Width	Midpoint of 2 to 4 to midpoint of 12 to 10
Mid Neuromast Width	13 to 14
Avg Length Olfactory to Mouth	Avg of 3 to 16 and 11 to 16
Area Top	Area of 2 to 12 to 10 to 4
Area Bottom	Area of 4 to 10 to 25 to 21
Area Combined	Area top + area bottom
Mid Olfactory to Chin Height	Midpoint of 3 to 11 to point 23
Mouth Area	
Mouth Perimeter	
Libiale Superius Angle	5 to 18 to 9
Chelion Left Angle	5 to 9 to 18
Chelion Right Angle	9 to 5 to 18
Chelion Angle Difference	Difference
Labiale Inferius Angle	17 to 7 to 19
Crista Philtri Left Angle	17 to 19 to 7
Crista Philtri Right Angle	19 to 17 to 7
Crista Philtri Angle Difference	Difference
Labiale Superius Mid Angle	6 to 18 to 8
Labiale Inferius Left Angle	6 to 8 to 18
Labiale Inerius Right Angle	8 to 6 to 18
Labiale Inferius Angle Difference	Difference

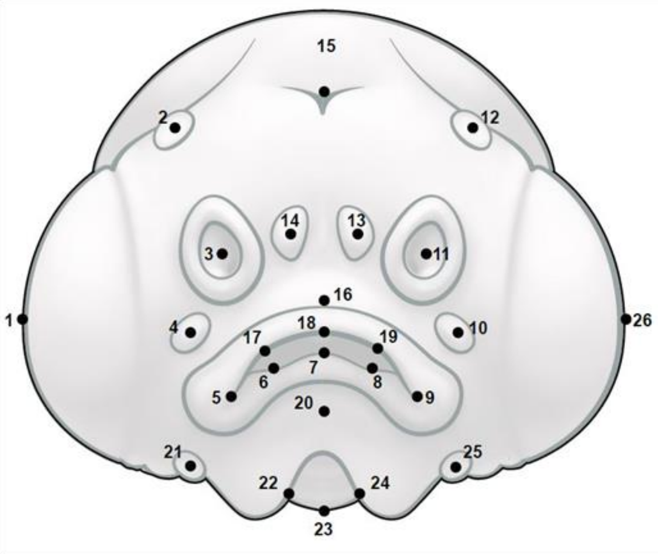

Supplemental Table 1. **zFACE analysis results at 6 dpf.** Shown in bold are parameters consistently altered at both 5 and 6 dpf.

zFACE feature	5dpf results	UIC vs fosMO	fosMM vs fosMO
**Width**	lower	***	***
Height		****	****
**Olfactory Distance**	lower	****	****
Upper Lip Width	higher		
Lower Lip Width	higher	****	**
**Mouth Width**	lower	****	****
**Olfactory to Mouth**	higher	****	****
Olfactory to Mouth 2	higher		
Difference		****	****
**Olfactory to Mouth 3**	lower	****	****
Chin Width	lower		
Mouth to Chin			
Alternate Height		****	****
**Mouth Height**	higher	****	****
**Neuromast Angle 1**	higher	****	****
**Neuromast Angle 2**	higher	****	****
Difference	higher		
Neuromast Height			
**Neuromast Width**	lower	****	****
**Mid Neuromast Width**	lower	***	*
Avg Length Olf mouth		****	****
Area Top	lower		
Area Bottom	lower		
Area Combined	lower		
Mid Olf. to Chin height		****	****
Mouth Area			
**Mouth Perimeter**	lower	****	****
**Libiale Superius Angle**	lower	****	****
**Chelion Left Angle**	higher	****	****
**Chelion Right Angle**	higher	****	****
**Chelion Diff**	bigger	****	****
**Labiale Inferius Angle**	lower	****	****
**Crista Philtri Left Angle**	higher	****	****
**Crista Philtri Right Angle**	higher	****	****
Crista Philtri Diff	higher		
**Labiale Superius Mid Angle**	lower	****	****
**Labiale Inferius Left Angle**	higher	****	****
**Labiale Inferius Right Angle**	higher	****	****
Labiale Inferius Diff			

Supplemental Table 2. **Fifth ceratobranchial (CB) arch mineralization and pharyngeal tooth development is affected in *fos* morphant embryos.** Pharyngeal teeth were counted in embryos at 5-8 dpf and tabulated the developmental timeline they appear (4V first and 4V2 last). While UIC and MM embryos had completely mineralized CB5s and developed most of the pharyngeal teeth, *fos* morphants showed only partially mineralized CB5s by 8dpf and failed to develop specific rows of teeth. UIC, uninjected control; MO, morphant; MM, mismatch morpholino control

Day post fertilization (dpf)	Tooth Id.	UIC		*fos*MM		*fos*MO	
		Left	Right	Left	Right	Left	Right
5dpf	5th arch	100%		100%		24%	
	4V	100	100	100	100	91	93
	3V	89	78	73	54	10	2
	5V	67	0	60	33	7	0
6dpf	5th arch	100%		100%		47%	
	4V	100	75	100	100	100	100
	3V	100	75	75	75	47	47
	5V	100	75	75	50	27	37
7dpf	5th arch	100%		100%		58%	
	4V	91	91	100	100	100	100
	3V	90	73	100	100	75	50
	5V	73	73	100	100	25	17
	4V2	64	55	88	88	0	0
8dpf	5th arch	100%		100%		69%	
	4V	100	100	100	100	93	100
	3V	100	86	100	100	69	66
	5V	86	86	100	100	41	45
	4V2	71	86	80	100	7	10
